# Isolation of viable monkeypox virus from anal and urethral swabs, Italy, May to July 2022

**DOI:** 10.2807/1560-7917.ES.2022.27.36.2200675

**Published:** 2022-09-08

**Authors:** Davide Moschese, Giacomo Pozza, Davide Mileto, Andrea Giacomelli, Miriam Cutrera, Maria Vittoria Cossu, Maddalena Matone, Martina Beltrami, Federica Salari, Spinello Antinori, Alessandra Lombardi, Giuliano Rizzardini

**Affiliations:** 1 I Division of Infectious Diseases, Luigi Sacco Hospital, ASST Fatebenefratelli Sacco, Milan, Italy; 2 III Division of Infectious Diseases, Luigi Sacco Hospital, ASST Fatebenefratelli Sacco, Milan, Italy; 3 Laboratory of Clinical Microbiology, Virology and Bioemergencies, Luigi Sacco Hospital, ASST Fatebenefratelli Sacco, Milan, Italy.; 4 Department of Biomedical and Clinical Sciences, Università degli Studi di Milano, Italy; III Division of Infectious Diseases, ASST Fatebenefratelli Sacco, Luigi Sacco Hospital, Milan, Italy.

**Keywords:** viral culture, polymerase chain reaction, viral isolation, sexual transmission

## Abstract

Anal and urethral samples from confirmed cases of monkeypox were screened for monkeypox virus (MPXV) by real-time PCR. Isolation of the virus was subsequently attempted in cell culture. Actively-replicating virus was demonstrated in 13 of 18 and 11 of 15 PCR-positive anal and urethral swabs, respectively, collected within 7 days from symptoms onset. Two asymptomatic secondary cases had detectable MPXV genetic material in urethral secretion and for one, MPXV was successfully isolated, supporting a potential MPXV sexual transmission hypothesis.

Since May 2022, a large outbreak caused by monkeypox virus (MPXV) is taking place, with, up to 15 August 2022, over 31,799 people having been infected with this virus in non-endemic areas [1]. This current outbreak involves human-to-human transmission, which may occur through contact with infectious material or infected people, including contact with skin lesions, or exposure to the respiratory droplets of a MPXV-infected individual during prolonged face-to-face contact [2,3]. These types of direct contacts can happen during intimate interactions between individuals, including sex [2,3]. MPXV genetic material has previously been identified in oropharyngeal and skin samples of monkeypox patients [4]. In addition, MPXV DNA has recently been detected in faeces, semen and urine from such patients, as well as in nasopharyngeal and rectal swabs [5-12]. We aimed to investigate the presence of live MPXV in anal and urethral swabs of patients with MPVX infection attending our sexual health clinic in Milan, Italy between 15 May and 7 July 2022.

## Screening for monkeypox virus DNA by PCR

Except for two individuals, all patients were screened for MPXV on clinical basis suspicion. Of the two people who were not tested on a clinical basis, one was completely asymptomatic, and the other reported 1 day of fever 3 days before the testing date. In this instance, the reason for testing was a reported close sexual contact with a laboratory-confirmed monkeypox case and the spontaneous request to be evaluated. 

Oropharyngeal and skin lesion swabs, as well as urethral and anal swabs were collected from suspected cases by means of Universal Transport Medium swabs (UTM-RT; COPAN Diagnostics, Italy). The samples were first investigated by a real-time PCR indiscriminately targeting variola virus and non-variola Orthopoxvirus species (RealStar Orthopoxvirus PCR Kit 1.0 – altona DIAGNOSTICS, Italy). Subsequently, samples testing positive by Orthopoxvirus PCR were further checked and confirmed for MPXV-DNA using an in-house real-time PCR specific for MPXV [5,13]. 

## Viral isolation

Urethral and anal specimens resulting positive for MPXV-DNA were used to attempt viral isolation. Briefly, a 200 μL aliquot of each sample (i.e. the transport medium in which the swab had been placed) was plated in duplicate in 24-well plates containing 80–90% confluent Vero E6 cells in 800 μL of Dulbecco's Modified Eagle Medium with l-glutamine (Gibco ThermoFisher Scientific) supplemented with 2% of heat-inactivated fetal bovine serum (Gibco ThermoFisher Scientific) and 1% penicillin‒streptomycin (5,000 U/mL; Pen-Strep, Gibco ThermoFisher Scientific). Plates were incubated at 37 °C and at 5% CO2 atmospheric pressure and checked every 24 hours. Cytopathic effect was observed in Vero E6 cells showing typical monolayer separation and cell rounding [13]; viral isolation was confirmed by Orthopoxvirus screening real-time PCR.

Signs of local infections in the adjacent areas of the anus and urethra were recorded during the physical examination to look at potential source of pre-analytic contamination. Patients with proctitis and rectal discharge were excluded from the present analysis in order to exclude potential MPXV contamination with lesional material.

## Characteristics of monkeypox cases

A total of 33 human monkeypox confirmed cases were enrolled. Epidemiological and clinical characteristics are presented in Table 1. All patients were self-identified men who have sex with men (MSM) with a median age of 38 years (interquartile range (IQR): 34─43). For 32 cases, at least one analysed anal swab was available, whereas all cases had at least one analysed urethral swab. 

**Table 1:** Monkeypox laboratory-confirmed-cases’ characteristics and delay between collection of anal and urethral swabs and time of symptom onset, Italy, May–July 2022 (n = 33 cases)

Characteristics	
Median age in years (IQR)	38 (34‒43)
Number of cases of male biological sex^a^	33
Number of cases with comorbidities other than HIV^b^	4
Number of cases who were people living with HIV	17
Number of cases with < 50 copies/mL of HIV-RNA	16
Median CD4 count (IQR)	678 (526‒933)
Number of cases on HIV PrEP	9
Number of cases with STIs in previous medical history	28
Number of cases with concomitant STIs	7
Number of cases with smallpox vaccination	3
Number of cases with self-reported sexual activity in the last month	
Number of cases with unprotected sexual intercourse	25
Number of cases with protected sexual intercourse only	5
Number of cases with oral unprotected intercourse only	3
Number of cases with specific lesions	
Number of cases with perianal lesions	7
Number of cases with lesions ≤ 2 cm from anal rim	6
Number of cases with glans lesions	3
Number of cases with at least one anal swab collected	32
Number of cases with anal swab collected ≤ 7 days from symptom onset	22
Number of cases with anal swab collected 8–14 days from symptom onset	7
Number of cases with anal swab collected 15–21 days from symptom onset	3
Number of days of cases with at least one urethral swab collected	33
Number of cases with urethral swab collected ≤ 7 days from symptom onset	18
Number of cases with urethral swab collected 8–14 days from symptom onset	13
Number of cases with urethral swab collected 15–21 days from symptom onset	2

CD4: cluster of differentiation; IQR: interquartile range; PrEP: pre-exposure prophylaxis; STIs: sexually transmitted infections.

^a^ Data on sex were collected as binary variables (male/female).

^b^ Major depression, anxiety disorder in two cases, previous hepatitis B and previous hepatitis C.

## Results of real-time PCR and viral culture

Individual longitudinal data comprehensive of RT-PCR quantitation cycle (Cq) values, results of viral culture and the time of viral culture positivity are presented for anal and urethral swab in Table 2. Actively-replicating virus in cell culture was demonstrated in 13/18 and 11/15 positive anal and urethral swabs, respectively, collected within 7 days from symptoms onset. For anal swabs, the longest positivity from symptom onset confirmed in viral culture was 16 days (Cq: 33) in a case, and for urethral swab this was 18 days (Cq: 28) in another case. One of the two cases belonged to people living with HIV; he had a well-controlled CD4 cell count of 933 cells/mm^3^ and for this case HIV-RNA was < 20 cp/mL (not detectable).

**Table 2:** Anal and urethral swabs, according to their collection time relative to symptom onset for each monkeypox case, with real-time-PCR results, viral culture results, and time to viral culture positivity, Italy, May–July 2022 (n = 33 cases)

Case number	Anal swab ≤ 7 days		Urethral swab ≤ 7 days		Anal swab 8–14 days		Urethral swab 8–14 days		Anal swab 15–21 days		Urethral swab 15–21 days	
	Days since symptom onset (Cq)	Viral culture (days to viral culture positivity)	Days since symptom onset (Cq)	Viral culture (days to viral culture positivity)	Days since symptom onset (Cq)	Viral culture (days to viral culture positivity)	Days since symptom onset (Cq)	Viral culture (days to viral culture positivity)	Days since symptom onset (Cq)	Viral culture (days to viral culture positivity)	Days since symptom onset (Cq)	Viral culture (days to viral culture positivity)
**1**	5 (17)	POS (3)	NA (NA)	NA (NA)	12 (24)	POS (4)	12 (34)	NEG (NA)	19 (38)	NEG (NA)	19 (NEG)	NA(NA)
**2**	4 (18)	POS (4)	4 (NEG)	NA (NA)	11 (28)	POS (5)	11 (NEG)	NA (NA)	NA (NA)	NA (NA)	NA (NA)	NA (NA)
**3**	NA (NA)	NA (NA)	NA (NA)	NA (NA)	NA (NA)	NA (NA)	8 (34)	NEG (NA)	16 (33)	POS (6)	16 (26)	POS (5)
**4**	NA (NA)	NA (NA)	NA (NA)	NA (NA)	14 (35)	Contaminated^c^	14 (NEG)	NA (NA)	NA (NA)	NA (NA)	NA (NA)	NA (NA)
**5**	4 (24)	Contaminated^c^	4 (34)	NEG (NA)	14 (NEG)	NA (NA)	14 (NEG)	NA (NA)	21 (NEG)	NA (NA)	21 (NEG)	NA (NA)
**6**	7 (20)	POS (4)	7 (19)	POS (4)	14 (36)	NEG (NA)	14 (NEG)	NA (NA)	21 (35)	NEG (NA)	21 (36)	NEG (NA)
**7**	3 (35)	NEG (NA)	3 (28)	POS (6)	NA (NA)	NA (NA)	NA (NA)	NA (NA)	21 (NEG)	NA (NA)	21 (NEG)	NA (NA)
**8**	NA (NA)	NA (NA)	NA (NA)	NA (NA)	NA (NA)	NA (NA)	10 (29)	POS (6)	NA (NA)	NA (NA)	NA (NA)	NA (NA)
**9**	NA (NA)	NA (NA)	NA (NA)	NA (NA)	13 (37)	Contaminated^c^	13 (NEG)	NA (NA)	19 (NEG)	NA (NA)	19 (37)	NEG (NA)
**10**	NA (NA)	NA (NA)	NA (NA)	NA (NA)	14 (NEG)	NA (NA)	14 (NEG)	NA (NA)	NA (NA)	NA (NA)	NA (NA)	NA (NA)
**11**	7 (31)	Contaminated^c^	7 (25)	POS (5)	NA (NA)	NA (NA)	NA (NA)	NA (NA)	NA (NA)	NA (NA)	NA (NA)	NA (NA)
**12**	2 (NEG)	NA (NA)	NA (NA)	NA (NA)	11 (NEG)	NA (NA)	11 (NEG)	NA (NA)	NA (NA)	NA (NA)	NA (NA)	NA (NA)
**13**	6 (16)	POS (3)	NA (NA)	NA (NA)	11 (21)	POS (4)	11 (34)	NEG (NA)	18 (NEG)	NA (NA)	18 (NEG)	NA (NA)
**14**	1 (23)	POS (5)	NA (NA)	NA (NA)	13 (NEG)	NA (NA)	13 (NEG)	NA (NA)	NA (NA)	NA (NA)	NA (NA)	NA (NA)
**15^a^**	3 (NEG)	NA (NA)	3 (35)	NEG (NA)	9 (NEG)	NA (NA)	9 (NEG)	NA (NA)	NA (NA)	NA (NA)	NA (NA)	NA (NA)
**16**	5 (34)	POS (7)	5 (35)	POS (7)	NA (NA)	NA (NA)	NA (NA)	NA (NA)	NA (NA)	NA (NA)	NA (NA)	NA (NA)
**17**	2 (34)	POS (7)	2 (30)	POS (6)	8 (NEG)	NA (NA)	8 (33)	POS (6)	NA (NA)	NA (NA)	NA (NA)	NA (NA)
**18**	7 (19)	POS (4)	7 (32)	NEG (NA)	8 (NEG)	NA (NA)	NA (NA)	NA (NA)	21 (NEG)	NA (NA)	NA	NA (NA)
**19^b^**	7 (NEG)	NA (NA)	7 (36)	POS (7)	14 (NEG)	NA (NA)	14 (NEG)	NA (NA)	NA (NA)	NA (NA)	NA	NA (NA)
**20**	NA (NA)	NA (NA)	NA (NA)	NA (NA)	11 (25)	POS (5)	11 (35)	NEG (NA)	21 (NEG)	NA (NA)	21 (NEG)	NA (NA)
**21**	7 (26)	POS (6)	7 (28)	POS (5)	14 (31)	NEG (NA)	14 (35)	NEG (NA)	18 (NEG)	NA (NA)	18 (36)	NEG (NA)
**22**	NA (NA)	NA (NA)	NA (NA)	NA (NA)	10 (NEG)	NA (NA)	10 (NEG)	NA (NA)	NA (NA)	NA (NA)	NA (NA)	NA (NA)
**23**	NA (NA)	NA (NA)	NA (NA)	NA (NA)	NA (NA)	NA (NA)	NA (NA)	NA (NA)	18 (NEG)	NA (NA)	18 (NEG)	NA (NA)
**24**	NA (NA)	NA (NA)	NA (NA)	NA (NA)	14 (22)	POS (5)	14 (NEG)	NA (NA)	21 (NEG)	NA (NA)	21 (NEG)	NA (NA)
**25**	6 (16)	POS (3)	6 (36)	NEG (NA)	13 (24)	POS (5)	13 (NEG)	NA (NA)	20 (35)	NEG (NA)	20 (NEG)	NA (NA)
**26**	3 (24)	POS (5)	3 (NEG)	NA (NA)	10 (NEG)	NA (NA)	10 (NEG)	NA (NA)	NA (NA)	NA (NA)	NA (NA)	NA (NA)
**27**	NA (NA)	NA (NA)	NA (NA)	NA (NA)	14 (30)	POS (6)	14 (NEG)	NA (NA)	NA (NA)	NA (NA)	NA (NA)	NA (NA)
**28**	3 (19)	Contaminated^c^	3 (31)	POS (6)	10 (17)	POS (4)	10 (25)	POS (5)	18 (NEG)	NA (NA)	18 (28)	POS (5)
**29**	4 (37)	NEG (NA)	4 (NEG)	NA (NA)	10 (NEG)	NA (NA)	10 (NEG)	NA (NA)	16 (NEG)	NA (NA)	16 (NEG)	NA (NA)
**30**	2 (NEG)	NA (NA)	2 (30)	POS (5)	11 (NEG)	NA (NA)	11 (28)	POS (5)	21 (NEG)	NA (NA)	21 (37)	NEG (NA)
**31**	NA (NA)	NA (NA)	NA (NA)	NA (NA)	NA (NA)	NA (NA)	NA (NA)	NA (NA)	18 (NEG)	NA (NA)	18 (NEG)	NA (NA)
**32**	7 (32)	POS (6)	7 (30)	POS (5)	14 (NEG)	NA (NA)	14 (37)	NEG (NA)	19 (NEG)	NA (NA)	19 (NEG)	NA (NA)
**33**	7 (31)	POS (6)	7 (25)	POS (5)	NA (NA)	NA (NA)	NA (NA)	NA (NA)	NA (NA)	NA (NA)	NA (NA)	NA (NA)

Cq: quantification cycle in real-time PCR; NA: not performed (either because there was no swab collected, or because the PCR was negative, so there was no subsequent viral culture); MPXV: monkeypox virus; NEG: negative; POS: positive.

^a^ The case was a variola-vaccinated contact of a confirmed monkeypox case who reported, 3 days before the swab collection, 1 day of fever. He did not develop rash nor any other symptom in the 28 days following the presumed date of MPXV exposure.

^b^ The case was vaccinated against variola and reported a 7-day-prior exposure to a confirmed MPX case. He did not develop rash in the 28 days following the presumed date of MPXV exposure. For this case the time to swab collection was calculated from the presumed date of MPXV exposure.

^c^ Viral culture contaminated by *Candida* spp. not allowing to assess the cytopathic effect of MPXV.

Two cases (cases 15 and 19) who did not develop rash within 28 days from the contact with a confirmed monkeypox case, had detectable MPXV DNA in the urethral swab. For one of these, the viral culture resulted positive. Both cases had oropharyngeal swabs testing negative in real-time PCR. Case 15 tested negative in real-time PCR on plasma also. Both cases had received one dose of variola vaccine (case 15 in 1984 and case 19 in 1973). Case 15 reported a presumed MPXV exposure 10 days before the swab collection and complained of only 1 day of fever 3 days before the sampling. Case 19 was tested 7 days after the exposure to a confirmed MPX case on his explicit request. He remained asymptomatic 28 days after the presumed date of MPXV exposure.

A graphic representation of the molecular and culture results obtained from anal and urethral swabs collected in monkeypox cases according to the elapsed time between symptoms onset and swab collection is presented in the Figure. The median PCR Cq of cases with a positive anal swab was 24 (IQR: 19–32) for those tested ≤ 7 days, 26 (IQR: 22–34) for those tested between 8 and 14 days and ranged from 33 to 38 for those tested between 15 and 21 days from symptom onset. The median PCR Cq of cases with a positive urethral swab was 30 (IQR: 28–35) for those tested ≤ 7 days, 34 (IQR: 29–35) for those tested between 8 and 14 days and ranged from 28 to 37 for those tested between 15 and 21 days from symptoms onset.

**Figure:**
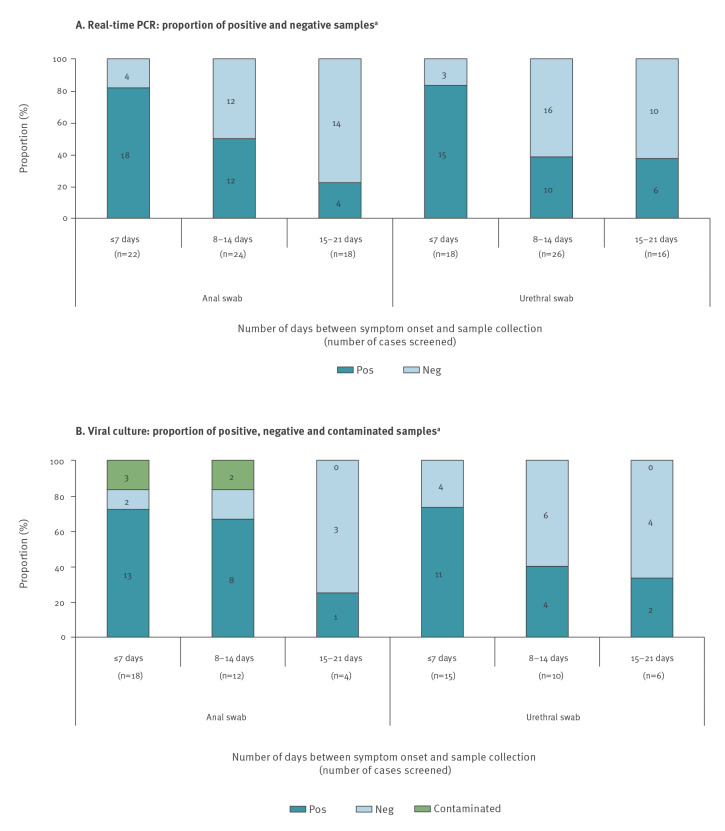
Graphical representation of the results of real-time PCR and viral culture derived from anal and urethral swabs from human monkeypox cases, according to time between symptom onset and swab collection, Italy, May–July 2022 (n = 33 cases)

Neg: negative; Pos: positive.

^a^ The numbers of cases screening positive, negative by PCR or viral culture, or with contaminated culture are on the chart bars.

The median PCR Cqs for positive anal swabs in which viable MPXV was and was not isolated were 24 (IQR: 19–30) and 35 (IQR: 35–37), respectively. The median Cqs for positive urethral swabs in which viable MPXV was and was not isolated were 28 (IQR: 25–31) and 35 (IQR: 34–36), respectively. No MPXV culture turned positive if Cqs above 34 and 36 had been found in anal and urethral swabs, respectively.

## Discussion

Monkeypox is endemic in Central and West Africa [14]. It is a viral zoonosis, however human-to-human transmission can also occur through contact with infected people [15]. Since May 2022, a large monkeypox outbreak in humans is affecting non-endemic areas. The clinical presentation of cases of this current outbreak is somewhat atypical, with, for example, the occurrence of lesions in the genital or perineal/perianal area and with some cases having no or delayed systemic symptoms [16,17]. So far, during the outbreak, cases of MPX have been primarily identified among groups of MSM [18].

A study from Nigeria published in 2020, reported genital manifestations of monkeypox in human cases and called for more investigations on the potential role of a sexual route in transmission [19]. Whether MPXV can be sexually transmitted, however, remains uncertain to date. While some studies have provided evidence of MPXV genetic material on rectal swabs by molecular methods, and viable virus in semen [12,20], these do not sufficiently support sexual transmission for several reasons. First, identification of MPXV DNA on a rectal swab does not imply the presence of replication-competent virus. Second, it is not known if sporadic detections of live MPXV in semen can be generalisable to all monkeypox male cases; moreover, the presence of live virus in semen does not necessarily imply that transmission can occur via semen, considering that a number of other viruses identified in semen do not seem to have direct evidence of sexual transmission [21].

In a previous monkeypox outbreak in the United States [22], which was caused by infected animals, the clinical manifestations of human monkeypox seemed to be influenced by the route of infection. Patients who had experienced non-invasive exposures (e.g. who touched an infected animal, as opposed to being bitten by one) appeared to have less pronounced signs of systemic illness and a milder course of disease. Some studies in animal models also suggest that the route of infection plays a role in disease presentation [23-25]. In this regard, the mild disease experienced by a large proportion of cases during the current outbreak and the absence of systemic symptoms in some of these cases, may point to non-invasive exposures, that could occur upon close or intimate contact with a MPXV infected individual.

To provide more evidence on whether viral transmission can occur by sexual contact, we analysed anal and urethral swabs from 33 confirmed human monkeypox cases and investigated whether viable virus was present. We found not only that the majority of both anal and urethral swabs collected within 7 days from symptoms onset tested positive for MPXV by PCR (18 PCR positive among 22 anal swabs and 15 PCR positive among 18 urethral swabs), but also that viable MPXV was present in most of the latter (13 culture-positive among 18 PCR-positive anal swabs and 11 culture-positive among 15 PCR-positive urethral swabs).

As expected, there appeared to be higher median Cq values for both anal and urethral swabs collected after the first week from symptom onset compared to within this week. It is noteworthy that only six cases had a perianal lesion located less than 2 centimetres from the anal rim and only three cases presented a glans lesion, thus making unlikely the potential pre-analytic contamination of our samples from surrounding lesions. It is also worth mentioning that two variola-vaccinated cases (who never developed rash) had respective urethral swabs with detectable MPXV DNA. For one of these cases, viable virus was recovered from the swab. This suggests that transmission from asymptomatic subjects is conceivable. 

To best of our knowledge this is the first identification of viable MPXV in anal and urethral swabs. We consider unlikely the possibility of contamination during sexual contact that could have led to false-positive results, given that the presumed times of last MPXV potential exposure in the concerned cases were 10 and 7 days before the swab collection. The results can be considered in terms of the hypothesis of a sexual-transmission potential of MPXV. Nevertheless, it is still unknown when the viral shedding starts in the anus and urethra after an infection, as well as the exact duration of viral shedding in such parts of the body; in our experience, an increase of Cq was observed from week 1 to week 3 post-symptom apparition in both anal and urethral samples, which appears to indicate a decline of the viral agent presence during the first 21 days from symptoms onset. As more data are necessary to affirm the timing of viral clearance, the authors support the US Centers for Disease Control and Prevention (CDC) and other Public Health institutions’ recommendation of condom use for several weeks after monkeypox recovery [26].

Our study has several limitations: (i) no systematic swab sampling was performed; (ii) although no cases reported rectal symptoms, we were not able exclude asymptomatic rectal lesions because no proctoscopy was performed; (iii) due to the limited size of our sample we were unable to correlate RT-PCR Cq with viral culture and we cannot provide a threshold to be used in clinical practice; (iv) given that the median Cq of a positive urethral swab was 30 (IQR: 28–35) for cases tested ≤ 7 days from symptom onset and 34 (IQR: 29–35) for those tested between 8 and 14 days from appearance of symptoms, the asymptomatic case, who underwent a urethral swab 10 days after MPXV exposure, and who had a Cq of 35, might have been borderline positive. On the other hand, samples from urethra from two other cases that had a Cq of 35 or more, tested positive for MPXV in cell culture.

In our study, the identification in swabs of viable MPXV, also in asymptomatic cases, indicates the possibility of a still unconfirmed sexual route of MPXV transmission. The presence of virus in anal swabs could justify the high incidence of MPXV infection in the MSM population, not necessarily because of engaging in risky sexual behaviours but due to the natural history and viral shedding of MPXV [27]. Once again, scientific findings are key to inform measures to prevent both disease transmission and stigma.
